# The Role of Glutamine and Glutaminase in Pulmonary Hypertension

**DOI:** 10.3389/fcvm.2022.838657

**Published:** 2022-03-02

**Authors:** Shang Wang, Yi Yan, Wei-Jie Xu, Su-Gang Gong, Xiu-Jun Zhong, Qin-Yan An, Ya-Lin Zhao, Jin-Ming Liu, Lan Wang, Ping Yuan, Rong Jiang

**Affiliations:** ^1^Department of Cardio-Pulmonary Circulation, Shanghai Pulmonary Hospital, Tongji University School of Medicine, Shanghai, China; ^2^Institute for Cardiovascular Prevention (IPEK), Ludwig Maximilian University of Munich, Munich, Germany; ^3^German Centre for Cardiovascular Research (DZHK), Partner Site Munich Heart Alliance, Munich, Germany; ^4^Department of Clinical Laboratory, Shanghai Pulmonary Hospital, Tongji University School of Medicine, Shanghai, China; ^5^Department of Respiratory Medicine, Yueyang Hospital of Integrated Traditional Chinese and Western Medicine, Shanghai University of Traditional Chinese Medicine, Shanghai, China; ^6^Department of Respiratory, Sijing Hospital of Songjiang District, Shanghai, China; ^7^Department of Respiratory and Critical Care Medicine, The First Hospital of Kunming, Kunming, China

**Keywords:** pulmonary hypertension, glutamine hydrolysis, glutaminase, tricarboxylic acid cycle, targeted therapy

## Abstract

Pulmonary hypertension (PH) refers to a clinical and pathophysiological syndrome in which pulmonary vascular resistance and pulmonary arterial pressure are increased due to structural or functional changes in pulmonary vasculature caused by a variety of etiologies and different pathogenic mechanisms. It is followed by the development of right heart failure and even death. In recent years, most studies have found that PH and cancer shared a complex common pathological metabolic disturbance, such as the shift from oxidative phosphorylation to glycolysis. During the shifting process, there is an upregulation of glutamine decomposition driven by glutaminase. However, the relationship between PH and glutamine hydrolysis, especially by glutaminase is yet unclear. This review aims to explore the special linking among glutamine hydrolysis, glutaminase and PH, so as to provide theoretical basis for clinical precision treatment in PH.

## Introduction

Pulmonary hypertension (PH) is a progressive and vicious vascular disease occurring via multiple mechanisms, which can lead to right heart failure as well as multiorgan dysfunction and is associated with a poor prognosis (1, 2). PH is often highly heterogeneous and has complex clinical manifestations. Epidemiological data on PH from several international registries show that the incidence of PH in the adult population is about 2.4 per million person-years and the prevalence is about 15 per million (3, 4). At the cellular and molecular level, PH is a complex panvasculopathy involving dysregulation of multiple vascular cell types, such as excessive proliferation, apoptosis resistance, accompanied by inflammation and fibrosis, which in turn results in the increase of pulmonary arterial pressure and the remodeling of right ventricular (RV) (5).

Pulmonary hypertension is classified by the World Health Organization (WHO) into five major categories based on the histopathology, hemodynamic characteristics, and possible etiology (6), such as pulmonary arterial hypertension (PAH), PH due to left heart disease, PH due to lung diseases and/or hypoxia, PH due to pulmonary artery obstructions, and PH with unclear and/or multifactorial mechanisms. Emerging evidence points out that no matter the type of PH, it ultimately shows a complex phenotype that can be observed in cancer characterized by excessive proliferation, resistance to apoptosis, epigenetic signature alteration and inflammation (7–10). As a consequence, the pathological changes akin to cancer cause the pulmonary vascular remodeling. Previous studies show that this phenotype switch is mainly related to the metabolic reprogramming *per se* (11, 12). Lines of evidence demonstrated that metabolic derangement might alter pulmonary vascular functions, and exacerbate symptoms, or reduce the survival rate of patients with PH (13). To date, the metabolic abnormalities manifested by the transition from oxidative phosphorylation to glycolysis, namely the Warburg effect, have become the limelight of metabolic studies in PH (14, 15).

However, this metabolic shift is not enough to meet the energy demand of the cells for excessive proliferation. The tricarboxylic acid cycle (TCA cycle) is very important for the synthesis of biological macromolecules. In TCA cycle, the corresponding intermediates of the cycle need to be continuously supplemented through the complementary pathway. In cancer, the hydrolysis of glutamine by glutaminase to provide intermediate products for TCA cycle is one of the most well-known complementary pathways (16). In recent years, the potential role of glutamine metabolism in the formation of PH has been increasingly discussed. Nevertheless, the exact contribution of glutamine metabolism, especially glutaminase, to PH is not fully understood. Herein, the review aims to summarize the glutamine metabolism, the role of glutamine and glutaminase on the progression of PH, so as to open a new avenue for PH treatment (7, 17).

## Properties of Glutamine and Glutaminase

Glutamine is the most abundant non-essential amino acid in the human body and synthesized from L-glutamic acid and ammonia through the action of the cytoplasmic enzyme glutamine synthetase (GS). Skeletal muscle is the main producer of plasma glutamine pool (18). Glutamine can be used as a raw material for biosynthesis when cells grow and divide. Carbon from glutamine is used in the synthesis of amino acids and fatty acids, and nitrogen from glutamine acts directly on the biosynthesis of purines and pyrimidines (19, 20).

Glutamine metabolism is a process in which cells convert glutamine into TCA cycle metabolites under the action of multiple enzymes. The first step in this process is the breakdown of glutamine into glutamate and ammonia by the action of glutaminase. There are two isozymes of glutaminase, one is kidney-type glutaminase (GLS1) located on chromosome 2 and the other is liver-type glutaminase (GLS2) located on chromosome 12. These two isozymes can produce multiple mutants by specific splicing (21). The role of these two enzymes has been well studied in oncology (22, 23). The activity of GLS is very high in small intestine, kidney, white blood cell and vascular endothelial cells (19). In addition, GLS1 is the main isoform expressed in cardiovascular tissues (17, 24). GLS1 has three isoforms: GLS (KGA, which is corresponding to longer transcript isoform), GLS C (GAC, which is corresponding to shorter transcript isoform) and GAM. GAM has no catalytic activity, while KGA and GAC differ only in C-terminal sequence. It was found that GAC has greater catalytic activity and is always upregulated than KGA in tumor cells (23). LGA (which is corresponding to shorter transcript isoform) and GAB (which is corresponding to longer transcript isoform). In most tumors, GLS2 serves as a tumor suppressor and GLS1 as an oncogene (25). The reason may be that GLS2 can bind to small GTPase Rac1 and inhibit its interaction with the Rac1 activator guanine nucleotide exchange factor, which in turn inhibits Rac1 and thus favoring the suppression of tumor metastasis (26). While GLS1 could interact with multiple regulatory factors such as MYC proto-oncogene (MYC), microRNAs and nuclear transcription factor-KB to promote tumor progression (21, 25). However, in some tumors, such as MYCN-amplified neuroblastoma tumor, GLS2 is highly expressed and promotes the occurrence and development of tumors (27).

To play a role, glutamine has to be transported by specific carriers on the cell membrane before the entry into the cell. Alanine-serine-cysteine transporter 2 (ASCT2), also known as solute carrier family 1, member 5 (SLC1A5) is one of the most important transporters in this process. Human ASCT2 is a Na^+^ dependent glutamine carrier located on the surface of cell membrane and lysosome membrane (28, 29). It is widely distributed in normal lung, skeletal muscle, large intestine, kidney, testis and brain (30).

## Glutamine Metabolism in Pulmonary Hypertension

The role of glutamine metabolism in tumor cells proliferation has been widely demonstrated. In malignant proliferating cells dominated by glycolysis, the upregulation of glutamine metabolism promotes energy metabolism via the modulation of TCA cycle and provides raw materials for lipid and amino acid delivery as well as biosynthesis of purines and pyrimidines, which ultimately promotes tumor growth by facilitating cell proliferation and inhibiting cell apoptosis (31, 32).

The upregulation of glutamine metabolism has been extensively studied in many types of cancer and it’s very critical for the pathogenesis of cancers, which renders it a well-known target for cancer therapy. The need for glutamine is particularly urgent in highly proliferating cells such as cancer cells and diseased blood vessel cells (5, 33). Growing evidence shows that GLS1 and GLS2 are related to tumor progression and growth rate, and tumor proliferation can be delayed by gene manipulation or inhibition/activation of these enzymes (34, 35). In concert with cancer metabolic switch for proliferating cells, the role of glutamine metabolism in the development of PH, especially glutaminase as a key enzyme in the initiation of glutamine hydrolysis pathway, has drawn great attention worldwide in recent years (17, 24). In this section, glutamine metabolism dysfunction, the mechanisms of glutamine metabolism mediated PH and key genes in regulation of glutamine metabolism in PH will be mainly discussed.

### Dysfunction of Glutamine Metabolism in Pulmonary Hypertension

Until now, metabolic reprogramming and mitochondrial dysfunction have been considered as the key events, leading to the excessive proliferation and anti-apoptosis of pulmonary vascular cells in the progress of PH (36, 37). Lines of evidence shows that amino acid metabolism, especially glutamine metabolism, plays an important role in tumorigenesis. For example, there exists a connection among the dysregulation of glutathione levels, tumor progression and cancer drug resistance (38, 39). Given the similarities between PH and cancer, studies were carried out to depict the relationship of glutamine metabolism and PH. Bertero et al. found that GLS expression was upregulated in lung tissues of human PAH (17). Similarly, Egnatchik et al. (33) demonstrated a systemic and pulmonary-specific alterations in glutamine metabolism, with the diseased pulmonary vasculature. In particular, bone morphogenetic protein receptor type 2 (BMPR2) mutation carriers had significantly more glutamine uptake than the control group. In addition, BMPR2 mutant pulmonary microvascular endothelial cells took up glutamine at twice the rate of WT cells, engendering a link between BMPR2 signaling and glutamine uptake. This team also identified that loss of sirtuin 3 (SIRT3) is a determinant for increased glutamine metabolism. On the contrary, preserving the function of SIRT3 is able to prevent the progress of PH in BMPR2 mutant mice via modulation of increased glutamine-driven metabolic reprogramming (33).

It is well known that structural and functional alterations of pulmonary arterial smooth muscle cells (PASMC) and pulmonary artery endothelial cells (PAEC) lead to remodeling of the pulmonary artery wall and increase vascular resistance. To address the glutamine alteration in those cells in PH, Bertero et al. (17) exposed PAEC and PASMC to a hard matrix and found increased uptake and breakdown of glutamine in both cell types, which may be related to activation of the YAP/TAZ-GLS1 axis and thus favoring a PH phenotype. As right ventricular failure is the main cause of death in PAH patients (40), how glutamine metabolism affect right ventricle also drew much attention in the setting of PH. It has been reported that the metabolic intensity of 14C-glutamine in the right ventricle of monocrotaline induced PAH rats was six-fold of that in the control rats (41). Accordingly, the expressions of glutamine transporters SLC1A5 and SLC7A5 were also upregulated in the right ventricle of monocrotaline induced PAH rats, which may be caused by the activation of the cMyc-Max pathway as a consequence of right ventricular ischemia. All these findings pinpoint a dysfunctional glutamine metabolism in PH.

### The Roles of Glutamine Metabolism Mediated Pulmonary Hypertension

**FIGURE 1:**
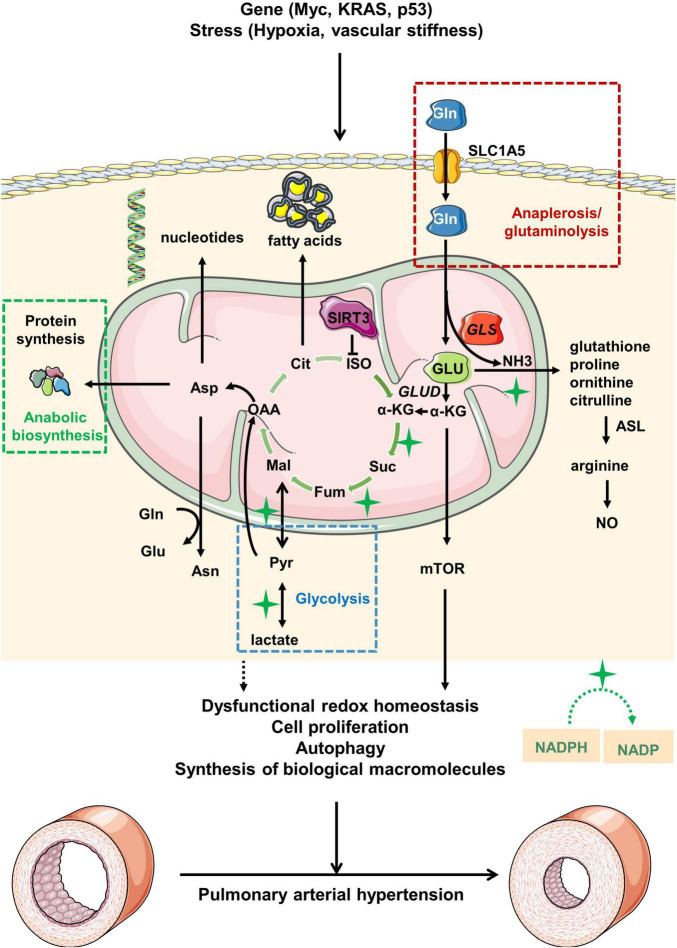
Overview of glutamine metabolism in pulmonary arterial hypertension. Under pathological stress or upon activation of oncogenes, glutamine (Gln) is transported from extracellular to intracellular space by alanine-serine-cysteine transporter 2 (ASCT2) [also known as solute carrier family 1, member 5 (SLC1A5)]. Gln is then hydrolyzed to glutamate (GLU) and ammonia (NH3) driven by increased GLS expression in mitochondria. GLU is transformed into α-ketoglutaric acid (α-KG), which participates in the tricarboxylic acid (TCA) cycle for energy supply. During the process, the abnormal glutamine metabolism would ultimately lead to the pathological changes of pulmonary arterial hypertension via modulation of redox homeostasis, cell proliferation, autophagy and synthesis of biological macromolecules. Myc, MYC proto-oncogene; KRAS, kirsten rat sarcoma viral oncogene; p53, p53 gene; GLUD, glutamate dehydrogenase; ISO, isocitrate; Cit, citrate; OAA, oxaloacetate; Mal, malate; Fum, fumarase; Suc, succinic acid; Asn, asparagine; Asp, aspartate; ASL, argininosuccinate lyase; NO, nitric oxide; Pyr, pyruvate; NADPH, nicotinamide adenine dinucleotide phosphate; NADP, nicotinamide adenine dinucleotide phosphate.

Glutamine metabolism is involved in the development of PH (Figure 1). Thus, exploring the role of glutamine metabolism in PH could lead to the discovery of novel therapeutic options in the management of pulmonary hypertension.

#### Promotion of Homeostasis of Redox

The synthesis of reactive oxygen species (ROS) and antioxidant glutathione (GSH) is essential in maintaining the redox balance in our body (42). Normally, physiological levels of ROS can maintain the homeostasis of redox, while excessive ROS would destroy the macromolecules within the body and lead to imbalance of redox. The transfer of electrons to oxygen via the mitochondrial electron transport chain to produce superoxide is one of the production pathways of ROS. During this process, glutamine metabolism plays an important role in maintaining intracellular ROS homeostasis. One of the most common pathways is that glutamine is metabolized to produce glutathione, which further affects ROS levels (19, 43). Studies have shown that the loss of GSH disrupts the redox homeostasis of cells, leading to the accumulation of ROS, which ultimately leads to cell damage and even death. At the same time, glutamine can also affect ROS homeostasis by producing NADPH through glutamate dehydrogenase (GLUD) (44). Besides, aspartic acid or malic acid produced by the TCA cycle is transported to the cytoplasm and converted by malate to pyruvate, during which NADPH is produced. The production of NADPH then provide the reducing equivalents for GSH reductase to regenerate GSH so as to promote redox homeostasis (45). NADPH-dependent transcriptional repressor C-terminal-binding protein 1 (CtBP1) was increased in fibroblasts from the pulmonary arteries of chronically hypoxic calves or idiopathic pulmonary arterial hypertension (IPAH) patients, which shaped the metabolic reprogramming of PH-fibroblasts toward glycolysis (46). All the findings implicate that dysfunctional glutamine metabolism might be responsible for the progression of PH via the manipulation of redox balance.

#### Promotion of Cell Proliferation

The phosphoinositide 3-kinase (PI3K)/AKT/mammalian target of rapamycin (mTOR) [PIK3/AKT/mTOR] pathway is one of the keys signaling pathways involved in cell proliferation (34), and activated by various growth factors and mitotic cytokines (34). The mTOR is a serine/threonine kinase and consisted of two different functional complexes, mTOR complex 1 (mTORC1) and mTOR complex 2 (mTORC2) (47). The mTORC1 is a major growth regulator that promotes cell proliferation in response to growth factors, extracellular nutrients and amino acids. The mTORC2 can promote cell survival by activating AKT, regulate cytoskeletal dynamics by activating protein kinase C, and control ion transport and cell growth by serum/glucocorticoid-induced kinase 1 phosphorylation (48). Tang et al. (49) found that two mTOR complexes play different roles in the development of PH. Inhibition of mTORC1 attenuated PH development. However, inhibition of mTORC2 results in a spontaneous increase in PH. In particular, amino acids, especially glutamine, play a crucial role in mTORC1 activation. Jenna et al. (50) pointed out that glutamine stimulates mTORC1 through a non-Rag GTPase-dependent mechanism. In RagA and RagB knockout cells, glutamine is still able to promote the transfer of mTORC to lysosome under the action of V-ATPase. Studies have also shown that PI3K/Akt1/mTOR signal pathway is involved in regulating the proliferation of pulmonary artery smooth muscle cells (PASMCs) in PH (51). mTOR-Notch3 signaling participated in chronic neonatal PH rat model after hypoxia exposure. Inhibition of mTOR or Notch3 is documented to prevent pulmonary artery remodeling after hypoxia challenge (52). In addition, glutathione metabolism is also dramatically altered in human PASMCs under the stimuli of the potent mitogen platelet derived growth factor-BB, a process regarded as a trigger for the media hypertrophy in pulmonary vasculature (9).

#### Regulation of Autophagy

Autophagy is an important process of intracellular turnover that is evolutionarily conserved in eukaryotes. In this process, some damaged proteins or organelles are wrapped by autophagic vesicles with double membrane structure and sent to lysosomes for degradation and recycling. In the process of autophagy, glutamine metabolism cannot be ignored (53, 54). Activated protein kinase 2 (GCN2) and integrated stress response (ISR) can induce autophagy, while glutamine can inhibit this process by inhibiting GCN2 activity and ISR (55, 56). In addition, glutamine can indirectly stimulate the mTOR pathway to inhibit autophagy (57). ROS as a stress response can also induce autophagy response, but glutathione and NADPH produced by glutamine metabolism can inhibit ROS production (58). Moreover, ammonia from glutamine catabolism can promote autophagy reactions through autocrine and paracrine (59). In addition, studies have shown that GS can directly regulate autophagy response by regulating glutamine metabolism (60). The abnormal autophagy regulation is associated with a variety of diseases, such as neurodegeneration, cancer, heart disease, liver disease, and vascular diseases (61, 62). In recent years, the role of autophagy in the development of PH has gradually become a research hotspot (63, 64). It is well known that pulmonary vascular remodeling is an important process in PH progression. Studies have found that autophagy abnormalities can be observed in PAECs, PASMCs and RV cardiomyocytes in animal models and patients with PH (63). Depending on the degree of autophagy activity, autophagy may be an inhibitor or promoter in the pathogenesis of PH.

#### Promotion of the Synthesis of Biological Macromolecules

Glutamine contains five carbon atoms, one amino nitrogen atom, and one amide nitrogen atom, which renders it raw materials for cell growth, division of biosynthesis. Carbon from glutamine is used in the synthesis of amino acids and fatty acids, and nitrogen from glutamine acts directly in the biosynthesis of purines and pyrimidines. It was found that glutamine-driven respiration in the mitochondria provides electron receptors for aspartic acid synthesis in proliferating cells, which in turn promotes nucleotide synthesis (20). Under the condition of hypoxia, the increased intake of glutamine promotes the synthesis of lipids from carbon atoms, which is a key process in malignant proliferating cells under stress (65). Glutamine promotes the synthesis of O-N-AcetylGlucosamine (GlcNAc) transferase (OGT) through the synthesis of uridine diphosphate O-N-AcetylGlucosamine (UDP-GlcNAc), which plays an important role in endoplasmic reticulum folding proteins (66, 67). Besides, study by Barnes et al. showed that OGT regulated the formation of vascular endothelial tube and the sprouting of vascular in idiopathic PAH, and inhibition of OGT also resulted in the decreased PASMCs proliferation (68).

### Common Genes Regulating Glutamine Metabolism in Pulmonary Hypertension

#### MYC Proto-Oncogene

MYC, as one of the common oncogenes in human cancer, is closely related to the upregulation of glutamine metabolism (69). It is reported that MYC mutated cells were significantly dependent on exogenous glutamine for the cell survival (56). MYC binds to promoter of high-affinity glutamine transporters (70), including ASCT2 and SN2 (also known as SLC38A5), and further upregulates the glutamine transporter, leading to increased glutamine uptake. Of note, MYC can also influence the activation of mTOR pathway by regulating the metabolic reprogramming of glutamine (71, 72). Although the specific contribution of MYC to the metabolic disturbance of PH has not been elucidated, it has been found that MYC activation is related to the proliferation of PAECs and PASMCs (5, 73). In addition, recombinant interleukin-6 (IL-6) treatment in rodent models under hypoxia led to pulmonary vascular remodeling, at least in part by MYC activation (74, 75). MYC was also proved to inhibit the expression of a microRNA family (mainly mir-23a and mir-23b) (5, 76), thereby increasing the expression of their target gene GLS and thus upregulating the glutamine catabolism, promoting the circulation of glutamine derived TCA cycle and glutathione production. Recently, it has been proposed that mir-23a can regulate PASMC proliferation and migration by regulating its target gene BMPR2 during the development of PH (77).

#### Kirsten Rat Sarcoma Viral Oncogene

The oncogene KRAS promotes the gene expression of enzymes related to glutamine metabolism in cells. Specifically, KRAS leads to the downregulation of GLUD, increases the dependence on glutamic-oxaloacetic transaminase 1 (GOT1), and releases aspartic acid into the cytoplasm through malic enzyme 1 (ME1) to produce NADPH, which ultimately increases the production of glutathione (70, 78). In addition, KRAS mutations can induce cell dependence on glutamine metabolism. However, this depends on different types of KRAS mutations. Compared with G12C and G12D mutant cells, the lung cancer tumor cells with KRAS-G12V mutation are less dependent on glutamine (79), but the specific mechanism is not clear at present. Pullamsetti et al. (80) described that KRAS transgenic mice exhibited an increased media thickness of small vessels and enhanced RV fibrosis in the tumor-bearing lungs, which mimicked the clinical manifestation of lung cancer-associated PH. However, the specific role of glutamine metabolism in this disease setting still needs to be further explored.

#### p53

Glutamine metabolism can also be regulated by tumor suppressor factors, such as p53, which contributes to gene defects repair and tumor stabilization. In transformed cells, the inhibition of malic enzymes ME1 and ME2 by p53 during the TCA cycle is essential for the production of NADPH and the metabolism of glutamine (81). The downregulation of ME1 and ME2 is also able to activate p53 mediated by protein kinases activated by AMP and MDM2 in a feed-forward manner. In addition, p53 can promote the expression of GLS2 and removes intracellular ROS to protect cells from DNA damage (26, 82). Besides, p53 promotes the expression of SLC1A3, an aspartic acid/glutamate transporter, allowing the use of aspartic acid to support cells in the presence of extracellular glutamine deficiency, thereby rendering tumor cells resistant to glutamine starvation (83). Hennigs et al. (84) demonstrated that p53 based angiogenesis therapy can activate the vasoprotective gene regulatory program to repair PAECs, regenerate pulmonary microvessels, and abolish PH. Recently, combination treatment by HIF-2α antagonist and p53 agonist has been proved to reverse established experimental PH (68). However, the interaction between glutamine metabolism, p53 and PH has not been fully described.

### Other Factors Linking Glutamine, Glutaminase and Pulmonary Hypertension

#### Hypoxia

Pulmonary hypertension is divided into five groups according to WHO PH classification. The third group is due to pulmonary disease and/or hypoxia, which accounts for almost a quarter of all patients with PH (85). Its pathophysiological mechanism involves hypoxic-related pulmonary vasoconstriction/remodeling, vascular endothelial and smooth muscle dysfunction, metabolomics derangements, inflammation, hypercoagulability, and so on (86–88). Hypoxia is considered to be a driver of metabolic conversion and glutamine metabolism in tumor cells (89). One of a recent publication also showed that glutamine and glutamate metabolism was the most remarkable altered metabolism in response to hypoxia in primary rat PASMCs (90). In addition, group 3 PH patients seems to take up more glutamine by the pulmonary vasculature compared to controls (33). HIF (including HIF1α and HIF2α) is a major transcription factor that tends to stabilize under hypoxia. Sun and Denko demonstrated that HIF1α stabilization inhibits glutamine oxidation (65), and HIF-2α may promote atypical glutamine metabolism by activating the PI3K/mTORC2 pathway during tumor progression (91).

#### Vascular Stiffness

Pulmonary sclerosis is an important component of the pathogenesis of PH, and stiffness can be used as an indicator of disease progression. The composition and quantity of the extracellular matrix (ECM), as well as vascular tension, can influence the vascular stiffness (5). The ECM network provides biophysical support for the various cells in the vascular wall, thus maintaining the mechanical stability and elastic recoil of the artery. Several pathogenic factors such as vascular injury, expression of pro-inflammatory factors, abnormal growth factors, and/or hypoxia exposure can cause ECM remodeling and stiffness (5). The accumulation of ECM is a significant pathological change in vascular wall in patients with PH. Recent studies have shown that arteriosclerosis and ECM remodeling are not only associated with end-stage PH, but also serve as early markers of PH (92, 93). The mechanotransduction of ECM refers to the process by which cells can perceive and adapt to external mechanical forces. However, studies on the process by which the mechanotransduction of is related to the vascular system are just emerging (17). The Hippo signaling pathway has two associated transcriptional co-activators, YES-associated protein 1 (YAP) and TAZ (or WWRT1), which are activated by the ECM and thus serve as a central regulator of cell proliferation, which can regulate the growth and development of tissue (94, 95). It was found that pulmonary vascular stiffness can activate YAP/TAZ at early PH, thus inducing the Mir-130/301 family to further enhance ECM remodeling and cell proliferation *in vivo* (96, 97). In addition, YAP/TAZ-induced pulmonary vascular stiffness has also been found to control important metabolic changes in PH (17, 92). In this process, YAP/TAZ is activated by the ECM in pulmonary vascular cell types, and YAP/TAZ subsequently activates the GLS enzyme, promoting glutamine metabolism and anaplerotic reaction. It also has downstream effects on cell proliferation, migration, and apoptosis among various vascular cell types in a time-and stage-specific manner, leading to changes in the extracellular environment that lead to pulmonary vascular dysfunction, which in turn causes PH (92).

## Targeted Therapy for Glutaminase in Pulmonary Hypertension

In recent years, glutamine metabolism has been regarded as one of promising therapeutic strategies against metabolic reprogramming in proliferative diseases such as tumors, PH and other cardiopulmonary diseases. Glutaminase, as a central part in glutamine metabolism, has emerged as a popular target (98, 99). Studies have shown that the expression of GLS in some breast cancer and nervous system tumors is higher than that in normal tissues, and the use of small molecule GLS inhibitors such as BPTES, CB-839 and compound 968 can significantly inhibit the growth of tumor cells (21, 100). Of note, whether GLS-targeted drugs can be applied in clinical practice is gradually attracting the attention. This section of the review will discuss the various drugs that target glutamine metabolism.

### Bis-2-(5-Phenylacetamido-1,2,4-Thiadiazol-2-yl)Ethyl Sulfide 3

Glutaminase exists in the form of dimer in an inactive state and is transformed into active tetramer after phosphorylation. BPTES are allosteric inhibitors of GLS, which can specifically bind to GLS and inhibit its phosphorylation activation (101, 102). In addition, BPTES can also inhibit the phosphorylation of GLS. Qie et al. (103) confirmed that BPTES could significantly downregulate or inhibit the proliferation of breast cancer cells with high GLS expression. Bertero et al. (17) observed that BPTES could inhibit the proliferation and migration of pulmonary vascular cells via modulation of the YAP/TAZ-GLS axis. However, the disadvantages of poor metabolic stability, low water solubility and low bioavailability limit the clinical application of BPTES (104).

### CB-839 (N-(5-(4-(6-((2-(3-(Trifluoromethoxy)Phenyl)Acetyl)Amino)-3-Pyridazinyl)Butyl)-1,3,4-Thiadiazol-2-yl)-2-Pyridineacetamide)

CB-839 is an oral GLS inhibitor developed on the basis of BPTES with a lower IC50 value (0.06 μM) and stronger effect compared to BPTES (101). CB-839 is a non-competitive inhibitor whose effectiveness does not depend on the concentration of glutamine. Drug metabolism associated studies have shown that CB-839 inhibits the activity of GLS, thereby restricting glutamine derivatives from entering the TCA cycle (105), so it has a significant inhibitory effect on the growth of breast cancer, soft tissue sarcoma and other malignant cells (106, 107). CB-839 is currently undergoing phase II clinical trials for Non-small Cell Lung Cancer (NSCLC) (Clinical Trial NCT02771626). Bertero et al. (17) showed that systemic administration of CB-839 alone was effective in improving PH in rats. However, Acharya et al. (108) showed that an inhaled form of CB-839 (without verteporfin) was not effective in alleviating PH. The divergency of the two studies might be due to the lower levels of CB-839 being administered via inhalation of PLGA particles.

### Compound 968 (5-(3-Bromo-4-(Dimethylamino)Phenyl)-2,2-Dimethyl-2,3,5,6-Tetrahydrobenzo[a]Phenanthridin-4(1H)-One)

Compound 968 is a selective non-competitive inhibitor of GLS, which has inhibitory effects on GLS1 and GLS2. It prevents the activation of GLS in cells by preventing the post-translational modification of GLS. Regarding on the inhibition of GLS, the allosteric inhibition mechanism of compound 968 is different from that of BPTES, which is characterized by different binding sites and mainly inhibits GLS in an inactive state. Compound 968 can inhibit the abnormal Rho-dependent signaling of GLS in tumor cells, thereby inhibiting the proliferation and migration of tumor cells, but has almost no effect on the proliferation of normal cells (102, 109). Its efficiency on the reversal of PH remains to be investigated.

### 6-Diazo-5-Oxo-L-Norleucine

6-diazo-5-oxo-L-norleucine competitively binds to the active site of glutamine and can form a covalent compound that irreversibly inhibits various glutamine metabolism-related enzymes such as GLS and GS, and produces analgesic, antiviral, and tumor inhibition effects (110, 111), alongside with strong side effects (111). In previous clinical trials for DON, it was found that high-dose intermittent administration and failure to screen for glutamine-dependent tumors are the main reasons for poor clinical effects and serious gastrointestinal reactions (112). Recently, people have gained a new understanding of the role of glutamine in a variety of tumor types, which has aroused people’s interest in metabolic inhibitors (such as DON). In particular, there have been many breakthroughs in the design of DON prodrugs, by modifying the carboxyl and amino groups of DON to obtain prodrugs that are not metabolized in plasma but decomposed in target cells, including acetyllysine substituted DON and JHU-083. At present, these two prodrugs have been studied in tumors. Studies have shown that acetyllysine substituted DON can be selectively metabolized in P493B lymphocytes and inhibit tumor cell proliferation in a dose-dependent manner (113). Leone et al. found that JHU-083 can not only inhibit the metabolism of glucose and glutamine in tumor cells, but also reverse the tumor microenvironment and maintain NADPH/NAD + homeostasis (114). Nevertheless, the reliable evidence demonstrating the efficacy of DON and its derivatives against PH is still lacking and warrants further investigation in this disease setting.

## Conclusion and Prospectives

Glutamine metabolism plays an important role in the proliferation and migration of tumor cells and vascular cells. Given the similarities between PH and cancers, the role of glutamine metabolism was explored in the development of pulmonary vascular remodeling. The dysfunctional glutamine metabolism leads to the excessive proliferation/migration of vascular cells via multiple mechanisms akin to that in tumor cells. A better understanding of glutamine metabolism mediated pulmonary vascular remodeling may be of great significance for the targeted therapy of PH. Glutaminase is a crucial enzyme in the process of glutamine hydrolysis and is also responsible for the modulation of glutamine metabolism. Although glutaminase inhibitors such as BPTES, CB839, Compound 968, and DON have been found to have significant inhibitory effects on many types of proliferating cells, its preclinical and clinical application to PH warrants further investigation to have more therapeutic gains.

## Author Contributions

RJ, PY, LW, and J-ML contributed to the conception of the article. Q-YA and Y-LZ searched the literature. SW, YY, and W-JX wrote the first draft of the manuscript. S-GG and X-JZ wrote the sections of the manuscript. All authors contributed to manuscript revision, read, and approved the submitted version.

## Conflict of Interest

The authors declare that the research was conducted in the absence of any commercial or financial relationships that could be construed as a potential conflict of interest.

## Publisher’s Note

All claims expressed in this article are solely those of the authors and do not necessarily represent those of their affiliated organizations, or those of the publisher, the editors and the reviewers. Any product that may be evaluated in this article, or claim that may be made by its manufacturer, is not guaranteed or endorsed by the publisher.
